# Hydrogen Peroxide and Hypochlorite Responsive Fluorescent Nanoprobes for Sensitive Cancer Cell Imaging

**DOI:** 10.3390/bios12020111

**Published:** 2022-02-11

**Authors:** Yun Chen, Jing Ye, Gang Lv, Weiwei Liu, Hui Jiang, Xiaohui Liu, Xuemei Wang

**Affiliations:** 1 State Key Laboratory of Bioelectronics, National Demonstration Center for Experimental Biomedical Engineering Education, School of Biological Science and Medical Engineering, Southeast University, Nanjing 210096, China; chenyun706@gmail.com (Y.C.); yej2017@163.com (J.Y.); liuw182@163.com (W.L.); sungi@seu.edu.cn (H.J.); lcswyh@126.com (X.L.); 2 Mathematical & Physical Science School, North China Electric Power University, Baoding 071003, China; glv7@ncepu.edu.cn

**Keywords:** hydrogen peroxide-triggered fluorescent nanoclusters, Ce6@Lum-AuNPs, FL nanoprobes, cancer cell bioimaging

## Abstract

Accurate diagnosis of cancer cells directly affects the clinical treatment of cancer and can significantly improve the therapeutic effect of cancer patients. Cancer cells have a unique microenvironment with a large amount of peroxide inside, effectively differentiated from relevant microenvironment normal cells. Therefore, designing the high-sensitive probes to recognize and distinguish the special physiological microenvironment of cancer cells can shed light on the early diagnosis of cancers. In this article, we design and construct a fluorescence (FL) contrast agent for cancer cell recognition and imaging analysis. Firstly, luminol-gold NPs (Lum-AuNPs) have been initially built, and then successfully loaded with the fluorescent receptor Chlorin e6 (Ce6) to prepare the luminescent nanoprobes (Ce6@Lum-AuNPs) with green synthesis, i.e., with biocompatible agents and mild temperature. The as-prepared fluorescent Ce6@Lum-AuNPs can efficiently and sensitively realize FL bioimaging of cancer cells. The relevant bio-sensing mechanism pertains to the presence of hypochlorite (ClO^−^); hydrogen peroxide (H_2_O_2_) in cancer cells could readily interact with luminol to produce chemiluminescence, which can activate the Ce6 component to emit near-infrared (NIR) FL. Therefore, this raises the possibility of utilizing the Ce6@Lum-AuNPs as efficient fluorescent nanoprobes for promising cancer early diagnosis and other relevant disease bioanalysis.

## 1. Introduction

In recent years, with the continuous progress of nanotechnology, more and more functional nanomaterials have been prepared, some of which have been widely used in biomedical diagnosis and treatment due to their excellent physical and chemical properties [1,2,3]. Nanomaterials, as new materials, are favored by researchers for their high surface-volume ratio unique mechanical and optical properties and are widely used in drug delivery, early diagnosis, and treatment of diseases [4]. Therefore, biomedical application based on nanomaterials has become a new trend in clinical research.

Bioimaging technology has made breakthrough progress in recent years. It has been widely and deeply applied in biomedical, clinical diagnosis, and other fields, supporting theoretical research and practical application [5]. Fluorescence (FL) bioimaging technology is the most widely used technology in optical bioimaging [6,7,8], which can realize real-time, dynamic, accurate, non-invasive monitoring of cancers and provide information on the dynamic changes of biological samples at the cellular and living level, which is favored by researchers and healthcare workers [9,10,11]. At present, more and more fluorescent probes are used in biological imaging technology, including quantum dots, upconversion materials, carbon-based materials, etc. [12,13,14]. However, many fluorescent probes have different degrees of cytotoxicity, stability, and resistance to photobleaching. For example, most quantum dots contain heavy metals such as cadmium and lead. Although the surface modification or the formation of a shell structure can reduce a certain degree of toxicity, frequently they cause cellular immune responses, etc. [15]. These deficiencies restrict their application in biomedicine.

Metal nanomaterials such as gold nanoparticles can effectively avoid the aforementioned defects and provide new choices and ideas for practical application. So far, there are relatively mature and large-scale chemical and physical methods for preparing metal nanomaterials, including hydrothermal synthesis, and microwave-assisted synthesis, ultrasonic synthesis, and so on [16,17,18,19,20,21]. Although these methods have some advantages, they also have the disadvantages of high environmental pollution, high cost, poor controllability, easy agglomeration of products, complex synthesis processes, and so on, which significantly restricts their further large-scale production and application in the biomedical field.

**Scheme 1:**
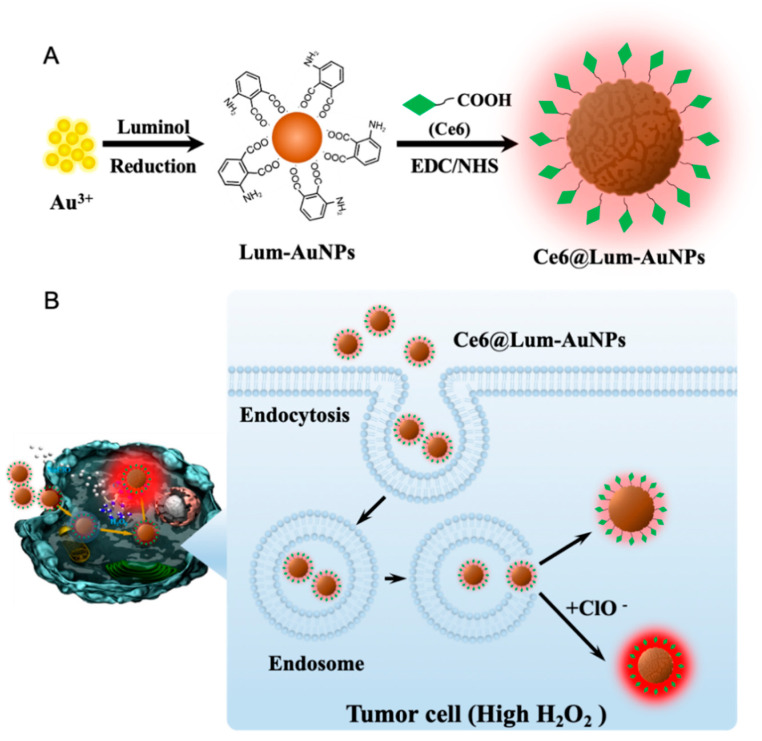
(**A**) Schematic diagram of green synthesis Ce6@Lum-AuNPs; (**B**) Schematic diagram of intracellular FL imaging of cancer cells.

Photosensitizers can also be used as FL imaging agents, and they are handy in diagnostic applications [22,23]. In a short, time excited singlet, photosensitizers can relax back to the ground state by emitting FL in the near-infrared (NIR) area of the spectrum [24]. The FL emission can be applied for disease location, photodynamic diagnosis, and molecular imaging, called photosensitizer FL detection (PFD) [25,26,27,28,29]. In this contribution, we have explored the possibility of designing and constructing Ce6@Lum-AuNPs with the synergetic enhancement of FL by H_2_O_2_ and ClO^−^, as the FL contrast agent for cancer cell recognition and imaging. AuNPs loaded with luminol and chlorin E6 (Ce6) have been developed as the luminescent nanoprobes to achieve FL imaging of cancer cells in situ (Scheme 1A). Luminol is luminescent with a maximum emission wavelength of 440 nm, and Ce6 is an FL receptor with a maximum absorption wavelength of 405 nm. Luminol units can interact with H_2_O_2_ in collaboration with ClO^−^, producing chemiluminescence and then activating the Ce6 component to emit near-infrared (NIR) FL. Therefore, Ce6@Lum-AuNPs can be used as an efficient fluorescent nanoprobe for FL imaging of cancer cells with high intracellular H_2_O_2_ levels, which lays a theoretical foundation for diagnosing cancers.

## 2. Experimental Section

### 2.1. Materials and Instruments

Materials: 1-(3-Dimethylaminopropyl)-3-ethylcarbodiimide hydrochloride (EDC), 5-Amino-2, 3-dihydro-1, 4-phthalazinedione (Luminol), and N-Hydroxysuccinimide (NHS) (Sigma-Aldrich, Saint Louis, MI, USA). Auric chloride acid (HAuCl_4_·4H_2_O) and dimethyl sulfoxide (DMSO) (Sinopharm Chemical Reagent Co., Ltd., Shanghai, China). 2-Amino-2-hydroxymethyl-propane-1, 3-diol (Tris) (Aladdin, Shanghai, China). Chlorin e6 (Ce6) (Shanghai Civi Chemical Technology Co., Ltd., Shanghai, China). 1,3-diphenylisobenzofuran (DPBF) (Shanghai Macklin Biochemical Co., Ltd., Shanghai, China). DMEM (high glucose) medium, streptomycin, penicillin, and trypsin (Hyclone, Logan, UT, USA). Fetal bovine serum (FBS, Ausgenex, Brissuspende, Queensland, Australia). Dialysis bag (cut-off = 1000 Da) (Spectrum Laboratories, Inc., Los Angeles, CA, USA). The water used in this study was prepared by Milli-Q (18.2 MΩ cm).

Instruments: BioMate 3S UV−Vis spectrophotometer (Thermo Scientific, Waltham, MA, USA). RF-5301 PC instrument (Shimadzu Co., Kyoto, Japan). JEM-2100 microscope (JEOL Ltd., Shimane, Japan). Thermo Scientific Nicolet iS5 instrument (USA). Microplate reader (Thermo Scientific, Waltham, MA, USA). Eclipse Ti2-E confocal laser scanning microscope (Nikon Instruments Inc., Tokyo Metropolis, Japan). LU-N4 Laser Units (Nikon Instruments Inc., Tokyo Metropolis, Japan).

### 2.2. Synthesis of Ce6@Lum-AuNPs

The AuNPs were synthesized by modifying our previously reported methods [30]. Luminol (10 mmol L^−1^, 3 mL) and HAuCl_4_ (50 mmol L^−1^, 600 μL) were mixed with the molar ratio of 1:1 in 5 mL Tris and left to set for at least 30 min at 25 °C. The AuNPs were collected by centrifuging the solution at 9600 g for 5 min and dispersed in deionized water under the ultrasonic wave. After four repetitions, the AuNPs were suspended in 1 mL DMSO by sonication dispersion. Then, the AuNPs were added to a mixture (containing 0.67 mg Ce6, 24 mg EDC and 12 mg NHS in 5 mL DMSO and stirred for 12 h at 37 °C in advance) for another 24 h stirring at 37 °C. Finally, the solution was purified by dialysis against water to remove unreacted reagents and by-products until no absorption was observed in the UV-Vis absorption spectrum. Finally, the drying of the solution by a vacuum oven at RT.

### 2.3. Ce6 Loading Capacity Calculation

The FL emission intensity at 667 nm (λ_ex_ = 405 nm) was taken for the Ce6 loading capacity calculation. Since the strong absorption peak of Lum-AuNPs is in the range of 275–450 nm, it is not suitable to use the absorbance of Ce6 at 405 nm to measure its load in nanoparticles. Therefore, detecting the FL emission spectra of Ce6 at different concentrations were exploited, and the relevant FL emission intensity at 667 nm (λ_ex_ = 405 nm) was determined, respectively. The standard curve for the relationship between the FL emission intensity and corresponding concentration was obtained by linear fitting. The related loading of Ce6 was calculated based on the standard curve.

### 2.4. FL Properties of the Ce6@Lum-AuNPs

An FL spectrometer was used to detect the FL properties of Ce6@Lum-AuNPs and the relevant changes of the FL properties in the presence of H_2_O_2_. In one group, 0.5 μL 100 mmol L^−1^ NaClO solution was added twice into 1 mL 2 μg mL^−1^ Ce6@Lum-AuNPs solution, the FL intensity—time curve was recorded. In another group, 1 mL 2 μg mL^−1^ Ce6@Lum-AuNPs solution was added with 1 μL10 mol L^−1^ H_2_O_2_ ten times, followed by adding 0.5 μL 100 mmol L^−1^ NaClO solution twice, the FL intensity—time curve was recorded as well. After the latter group of the solution was left at room temperature for a week, horseradish peroxidase (HRP) was added for photographs.

An amount of 0.5 μL 100 mmol L^−1^ NaClO solution was added twice into 1 mL Ce6@Lum-AuNPs solution with a concentration of 2 μg mL^−1^, and the FL emission spectrum was detected after each addition. Add 10 μL 10 mol L^−1^ H_2_O_2_ to 1 mL Ce6@Lum-AuNPs solution at a concentration of 2 μg mL^−1^. Then 0.5 μL 100 mmol L^−1^ NaClO solution was added twice, and the FL emission spectrum was detected after each addition. Finally, HRP was added to the latter solution to detect the FL emission spectrum.

### 2.5. Specificity of the Ce6@Lum-AuNPs for H_2_O_2_ and ClO^−^

The FL intensity of Ce6@Lum-AuNPs with and without H_2_O_2_ and NaClO in the presence or absence of various ROS, such as 50 μmol L^−1^ CO^+^, O_2_^−^, ^1^O_2_, OH· and ONOO^−^ was collected by FL emission spectra.

### 2.6. Cell Culture

HepG2 cells (Shanghai Institute of Biological Sciences, Chinese Academy of Science, Shanghai, China). L02 (human embryo liver cell strand) (Third Military Medical University, Chongqing, China). The cell was cultured in a culture medium (DMEM with 10% FBS and 1% streptomycin/penicillin).

### 2.7. In Vitro Cytotoxicity Test

*The* cytotoxicity test was performed by conventional CCK-8 assays. HepG2 and L02 cells were seeded in 96-well plates at 5 × 10^3^ cells for each well. After a 24 culture, the cells were rinsed and added 100 μL DMEM with various concentrations (40, 80, 160, 320, 640 μg mL^−1^) of Ce6@Lum-AuNPs as test groups (five wells for each group). Cell-free wells adding 100 μL DMEM with the same concentrations of Ce6@Lum-AuNPs were used as control groups. After 24 h continuous incubation, 10 μL of CCK-8 solution was added and incubated for another 1 h. The suspensions were measured at 450 nm. According to the improved Karber’s method, IC_50_ values were calculated.

### 2.8. In Vitro Bioluminescence Imaging of Cancer Cells

HepG2 and L02 cells were added into a confocal petri dish with a concentration of 5 × 10^3^ cells for each dish. The cells were placed in an incubator containing 5% CO_2_ humidification at 37 °C overnight. After culture, the cells were washed with PBS; then the control group was added 2 mL fresh medium with nothing. One experimental group added 2 mL of new medium containing 1.35 μg mL^−1^ Ce6. Two other groups were added with fresh medium containing 50 μg mL^−1^ Ce6@Lum-AuNPs. After culturing for 6 h, 0.5 μL 100 mmol L^−1^ NaClO was added into one of the 50 μg mL^−1^ Ce6@Lum-AuNPs groups. After further cultivation for 4–6 h, the FL distribution in cells was observed under a confocal laser FL microscope.

## 3. Results and Discussion

### 3.1. Synthesis and Characterization of Ce6@Lum-AuNPs

**Figure 1:**
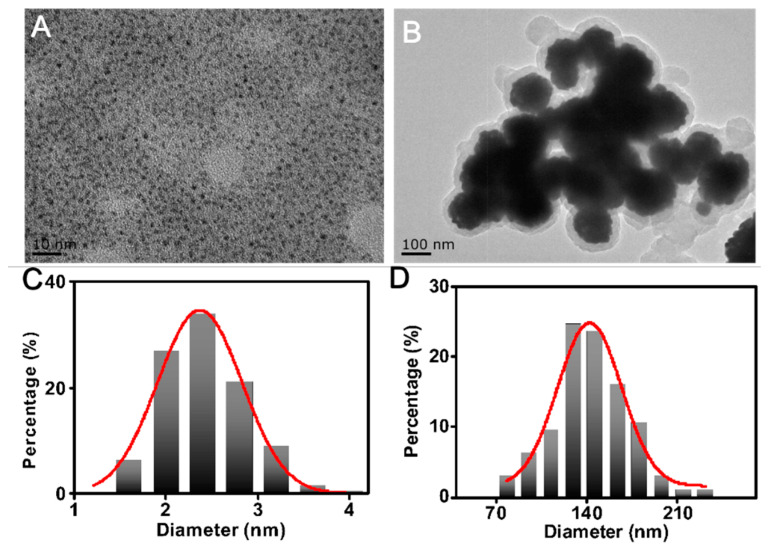
(**A**) TEM image of Lum-AuNPs, (**B**) TEM image of Ce6@Lum-AuNPs, (**C**) Size distribution curves of Lum-AuNPs; (**D**) Size distribution curves of Ce6@Lum-AuNPs.

According to the synthesis method and optimization procedure reported in the literature [30], luminol was used as a reducing agent to reduce HAuCl_4_ to AuNPs through redox reaction and luminol coated gold nanoparticles (Lum-AuNPs) were prepared. TEM images showed that Lum-AuNPs and the core of Ce6@Lum-AuNPs had an average size of approximately 2.3 ± 0.4 nm and 143.8 ± 29.7 nm, respectively (Figure 1). Dynamic light scattering results showed that Ce6@Lum-AuNPs were well-dispersed in water, with a mean hydrodynamic size of 183.9 ± 6.6 nm and a polydispersity index of 0.136.

**Figure 2:**
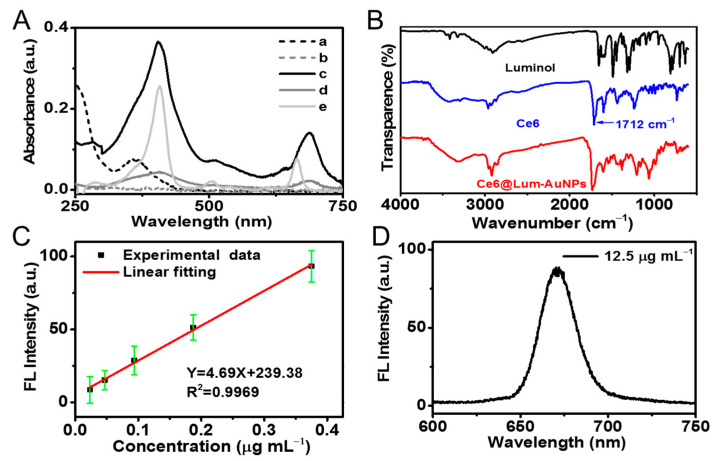
Synthesis and characterization of Ce6@Lum-AuNPs. (**A**) UV-Vis absorption spectra of Ce6@Lum-AuNPs before and after dialysis. (a and b) Dialyzed-out solution for the first and last dialysis procedures, respectively. (c) Original Ce6@Lum-AuNPs solution in DMSO. (d) Dialyzed Ce6@Lum-AuNPs solution in water. (e) Ce6 at the concentration of 0.94 μg mL^−1^. (**B**) FT-IR spectra of luminol, Ce6, and Ce6@Lum-AuNPs. (**C**) Calibration curve of the FL emission intensity of Ce6 at 667 nm and corresponding concentration. (**D**) FL emission spectra and intensities of Ce6@Lum-AuNPs at 12.5 μg mL^−1^ (λ_ex_ = 405 nm).

UV-Vis absorption spectra (Figure 2A) were used to monitor the dialyzed-out solution (curves a and b). After dialysis, the UV-Vis absorption spectrum of the prepared Ce6@Lum-AuNPs showed two characteristic peaks at 409 nm and 689 nm, respectively (curve e). Compared with Ce6, the UV-Vis absorption spectrum of Ce6@Lum-AuNPs showed significant redshift (curves c and d). The FT-IR spectra showed that the surface of Lum-AuNPs was functionalized with -NH_2_ group by 3-aminophthalic acid (Figure 2B). Then Ce6 was conjugated on the Lum-AuNPs by an amide condensation reaction to obtain the Ce6@Lum-AuNPs. In addition, the FT-IR spectrum of Ce6@Lum-AuNPs showed that characteristic absorption bands of Ce6 and Luminol were detected. These results indicated that luminol and Ce6 were successfully modified on the AuNPs surface.

The FL intensity of Ce6 with different concentrations at 667 nm was measured, and the standard curve between FL intensity and concentration was drawn in Figure 2C. Then, based on the emission intensity of Ce6@Lum-AuNPs at 667 nm (λ_ex_ = 405 nm), the bearing capacity of Ce6 was calculated to be 2.7% (mass ratio) according to the standard curve.

### 3.2. FL Spectroscopy Characterization of Ce6@Lum-AuNPs

**Figure 3:**
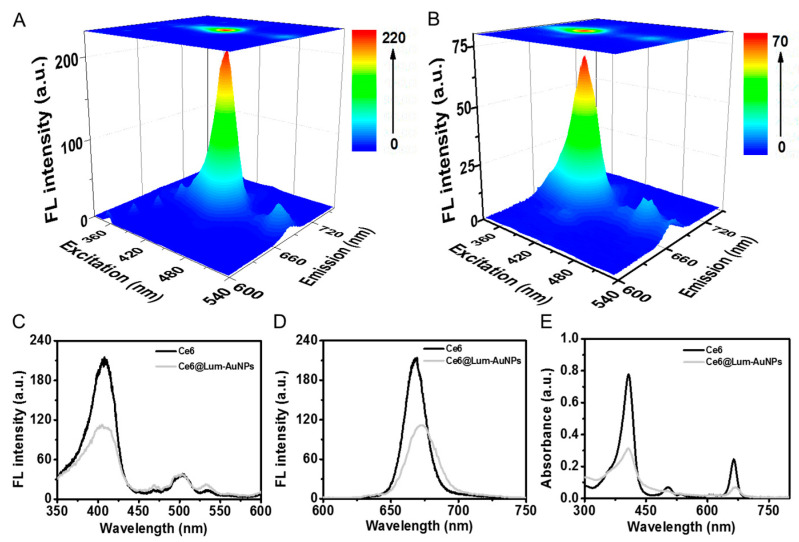
Spectroscopy characterization of the Ce6@Lum-AuNPs. (**A**,**B**) 3D FL spectra of Ce6@Lum-AuNPs (**A**) and Ce6 (**B**) in DMSO. (**C**,**D**) FL excitation (**C**) and FL emission (**D**) spectra of Ce6@Lum-AuNPs and Ce6 in DMSO. (**E**) UV-Vis spectra of Ce6@Lum-AuNPs and Ce6 in DMSO.

The emission spectra of Ce6 and Ce6@Lum-AuNPs were recorded at different excitation wavelengths, and three-dimensional (3D) FL spectra (Figure 3A,B) were also illustrated. It could be shown that the emission spectra of Ce6@Lum-AuNPs and Ce6 have relevant similar spectroscopic characteristics. FL excitation spectra of Ce6 and Ce6@Lum-AuNPs in DMSO were shown in Figure 3C, and the maximum excitation wavelength for both was around 400 nm. Figure 3D showed the FL emission spectra of Ce6 and Ce6@Lum-AuNPs, indicating that the maximum emission wavelength of Ce6@Lum-AuNPs was 670 nm, showing an apparent red shift compared with that of Ce6 at 660 nm. Figure 3E showed that the UV-Vis absorption spectrum of Ce6@Lum-AuNPs was similar to that of Ce6, which again illustrates that Ce6 was successfully loaded on gold nanoparticles. Based on the above observations, Ce6@Lum-AuNPs with NIR FL emissions were successfully obtained and had similar spectroscopic properties to Ce6.

**Figure 4:**
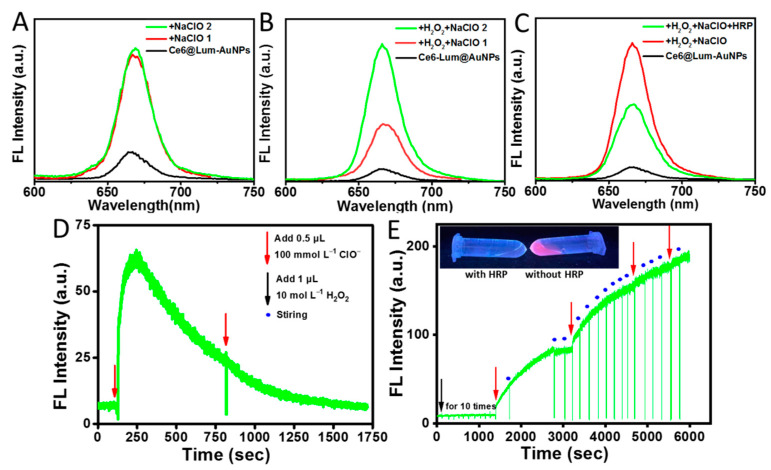
FL changes properties of Ce6@Lum-AuNPs with different treatments. (**A**,**C**) FL emission spectra of Ce6@Lum-AuNPs with different treatments (**A**) add NaClO; (**B**) add H_2_O_2_ and NaClO successively; (**C**) add HRP in Ce6@Lum-AuNPs include H_2_O_2_ and NaClO solution. (**D**,**E**) FL intensity-time curves of Ce6@Lum-AuNPs under different treatments. Inset: FL photos of Ce6@Lum-AuNPs (including H_2_O_2_ and NaClO) under UV lamp taken after one week with and without HRP addition.

Considering the relatively high concentration of H_2_O_2_ in the intracellular microenvironment of cancer cells, the effect of H_2_O_2_ on the FL properties of Ce6@Lum-AuNPs was investigated for the possibility to apply it for cell recognition and bio-imaging further. Relevant FL emission spectra under different treatment conditions were detected, as shown in Figure 4A. It was evident that when NaClO was added to Ce6@Lum-AuNPs aqueous solution, the fluorescence intensity was the same for the first time and the second time, but both were stronger than that without NaClO. Figure 4B showed that after Ce6@Lum-AuNPs aqueous solution was pre-mixed with H_2_O_2_, the FL intensity of the solution with two NaClO additions was significantly enhanced compared to that with only one addition. As shown in Figure 4C, after adding HRP, the FL emission intensity of the solution decreased significantly. Hypochlorous acid with high reactivity is generated by the neutrophil enzyme myeloperoxidase and is particularly relevant in inflammatory conditions. Meanwhile, hydrogen peroxide is a strong two-electron oxidant, but most of its electron oxidation reactions are too slow to be biologically relevant because of high activation energy. NaClO is applied to simulate the tumor tissue microenvironment and oxidate luminol in collaboration with H_2_O_2_ [31].

The change of FL intensity was collected by FL intensity-time curves with an excitation at 405 nm. After adding NaClO to Ce6@Lum-AuNPs aqueous solution, the FL intensity at 670 nm was significantly enhanced, then quickly decreased to the original level (Figure 4D). When NaClO was added again, the FL intensity did not change (Figure 4D). When the same aqueous solution was mixed with a certain amount of H_2_O_2_ solution beforehand, the FL intensity increased rapidly after adding NaClO (Figure 4E). Unlike the solution without H_2_O_2_, the FL intensity increased with added NaClO and was maintained for a week without a significant decrease (Figure 4E inset). It was considered that the addition of H_2_O_2_ changes the FL enhancement process. To verify the conjecture, horseradish peroxidase (HRP), which could hydrolyze hydrogen peroxide, was added into the latter solution. As could be seen under the UV lamp, the FL of the solution was significantly reduced, shown in the inset of Figure 4E. These observations were consistent with the results of FL emission spectra, indicating that the addition of H_2_O_2_ could readily facilitate the FL enhancement effect of NaClO on Ce6@Lum-AuNPs.

**Figure 5:**
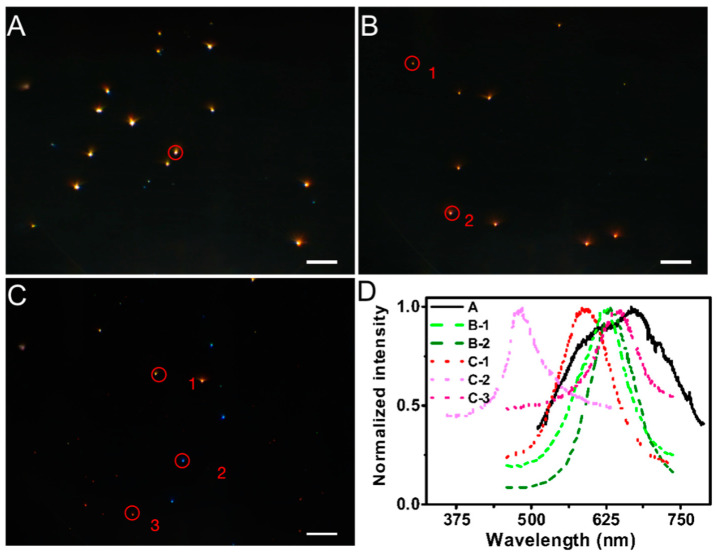
(**A**) DFM image of Ce6@Lum-AuNPs dispersed in an aqueous solution. (**B**) DFM image of Ce6@Lum-AuNPs solution treated with NaClO. (**C**) DFM image of Ce6@Lum-AuNPs solution treated with H_2_O_2_ and NaClO in order. (**D**) The DFM scattering spectra of a single nanoparticle marked in A-C. The exposure time for image and spectra were 100 ms and 1 s, respectively. The scare bar in DFM images was 5 μm.

Darkfield microscopy (DFM) was used to further observe the change of Ce6@Lum-AuNPs before and after H_2_O_2_ and NaClO treatment. Figure 5A–D were DFM images, and Figure 5D was the DFM scattering spectrum of a single nanoparticle marked with circles in the figures. As shown in the figures, Ce6@Lum-AuNPs without treatment were yellow in the DFM image, and their scattering spectrum had a broad peak. The particles treated with NaClO appear red in the DFM image, and their spectra peaks were narrow at about 625 nm. Finally, three kinds of particles with different scattering spectra appeared in the solution treated with H_2_O_2_ and NaClO, and the peaks of scattering spectra were 478 nm, 587 nm and 645 nm, respectively. The results showed that H_2_O_2_ and NaClO changed the surface structure of Ce6@Lum-AuNPs, contributing to the effects observed in relevant FL spectra.

### 3.3. Specificity of the Ce6@Lum-AuNPs for H_2_O_2_ and ClO^−^

**Figure 6:**
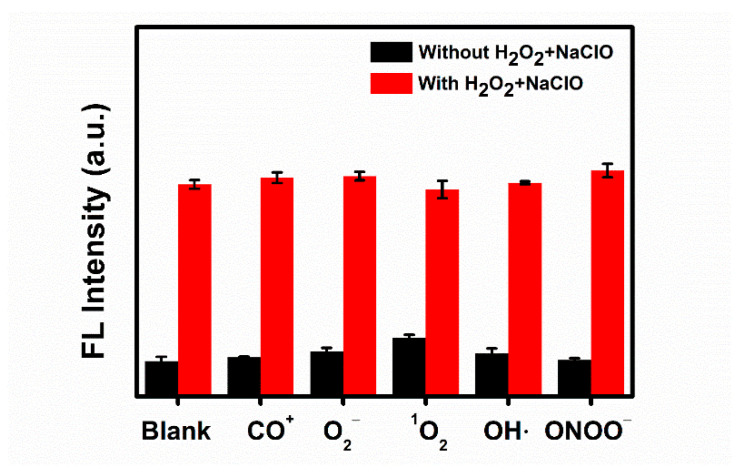
Study on the relevant FL signal changes of Ce6@Lum-AuNPs with and without H_2_O_2_ and NaClO in the presence or absence of CO^+^, O_2_^−^, ^1^O_2_, OH· and ONOO^−^.

Considering that there may exist other kinds of ROS in cells, we had investigated the specificity of the Ce6@Lum-AuNPs for H_2_O_2_ and NaClO. As shown in Figure 6, comparing the FL intensity of Ce6@Lum-AuNPs with and without H_2_O_2_ and NaClO in the presence or absence of CO^+^, O_2_^−^, ^1^O_2_, OH· and ONOO^−^, the results illustrated that the presence of these ROS had little or almost no influence on the relevant FL intensity. Meanwhile, our experimental results also indicated that there were few or no fluorescent changes observed upon addition of different concentrations of H_2_O_2_ alone to the solution of Ce6@Lum-AuNPs. Thus, it is hard to obtain the corresponding concentration for 50% of maximal effect (EC50) of Ce6@Lum-AuNPs for H_2_O_2_. Nevertheless, in the presence of hypochlorite (ClO^-^), hydrogen peroxide in cancer cells could readily interact with luminol to produce remarkable chemiluminescence (as described later). This raises the possibility of utilizing the relevant hydrogen peroxide and hypochlorite responsive fluorescent nanoprobes for sensitive cancer cell imaging.

### 3.4. Cytocompatibility Evaluation In Vitro

**Figure 7:**
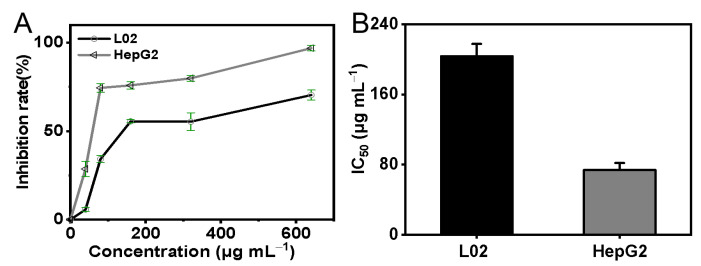
(**A**) The cells inhibition with different concentrations of Ce6@Lum-AuNPs. The error bars of each column show the standard deviation among three independent measurements. (**B**) The half-maximal inhibitory concentration (IC_50_) values of different cells (including HepG2 and L02 cells) after incubation with various doses of Ce6@Lum-AuNPs for 24 h.

The CCK-8 assays were used to evaluate the cytotoxicity in vitro. Both HepG2 and L02 cells were co-incubated with Ce6@Lum-AuNPs at different concentrations for 24 h. The cell inhibition rates of both cell lines increased with Lum-AuNCs concentration. For each concentration of Ce6@Lum-AuNPs, the inhibition rate of HepG2 cells was higher than that of L02 cells. The half-maximal inhibitory concentration (IC_50_) of Ce6@Lum-AuNPs for HepG2 and L02 cells were 73.76 μg mL^−1^ and 203.64 μg mL^−1^, respectively, determined by improved Karber’s method. The results showed that compared with L02 cells, Ce6@Lum-AuNPs had a relatively more substantial inhibitory effect on HepG2 cells (Figure 7).

### 3.5. High-Resolution FL Imaging of Cells In Vitro

**Figure 8:**
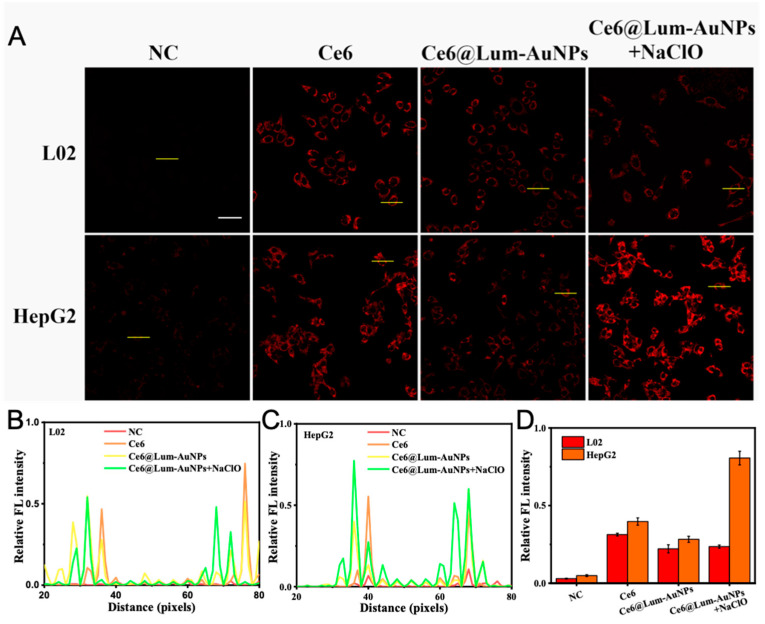
(**A**) Confocal FL imaging of Ce6@Lum-AuNPs and Ce6 incubated with HepG2 and L02 cells for 24 h, scale bar: 50 μm. (**B**–**D**) The relative FL intensity of the corresponding cell cross-sections (yellow lines) of L02 and HepG2 was treated differently.

For better application in biomedical imaging, we further examined the FL characteristics of Ce6@Lum-AuNPs in cells. Both Ce6@Lum-AuNPs and Ce6 were incubated with HepG2 and L02 cells for 12 h, respectively, and their FL properties and distribution in cells were observed under a confocal FL microscope. As shown in Figure 8A, no obvious FL signal was observed in cancer cells and normal cells in the control group without the addition of Ce6@Lum-AuNPs and Ce6. Both cancer cells and normal cells incubated with Ce6 showed an obvious FL signal due to the good FL performance of Ce6, but it could not distinguish cancer cells from normal cells. The Ce6@Lum-AuNPs group had a red FL signal in normal cells, while the FL signal in cancer cells was slightly stronger than normal cells. After the addition of NaClO, the FL signal in the Ce6@Lum-AuNPs group was significantly higher than that in the normal cells, which may be due to the special microenvironment in the cancer cells, such as a certain amount of H_2_O_2_, enhancing the FL. The relative FL intensities of L02 and HepG2 cell cross-sections were shown in Figure 8B–D. Therefore, we believe that Ce6@Lum-AuNPs can be used as a potential fluorescent contrast agent in cancer cells FL imaging studies.

## 4. Conclusions

In summary, we have explored the possibility of designing and constructing fluorescent Ce6@Lum-AuNPs as the FL contrast agent for cancer cell recognition and imaging analysis. Firstly, luminol-gold nanoparticles (Lum-AuNPs) have been readily built, and then successfully loaded the fluorescent receptor Ce6 to prepare the FL nanoprobes (Ce6@Lum-AuNPs) with green synthesis, i.e., with biocompatible agents and mild temperature. The as-prepared fluorescent Ce6@Lum-AuNPs can efficiently and sensitively realize FL bioimaging of cancer cells. The relevant bio-sensing mechanism is pertaining to the presence of the relatively high concentration of hydrogen peroxide in cancer cells that can readily interact with luminol in collaboration with ClO^−^ to produce bright chemiluminescence and thus activate the Ce6 component to emit NIR FL. Therefore, this novel strategy of self-luminescent nanoprobes is developed to achieve highly sensitive fluorescent bioimaging of cancer cells in situ. Using the microenvironment of cancer cells, NIR FL can be successfully emitted for the precise cancer cell image, laying a promising way for the accurate early diagnosis and treatment of cancers.

## Data Availability

The data presented in this study are available in article.
